# Molecular insights into seasonal trematode infections in *Bithynia* Snails: host lineages, parasite diversity, and *Opisthorchis viverrini* susceptibility in southern Lao PDR

**DOI:** 10.1186/s41182-025-00834-1

**Published:** 2025-11-26

**Authors:** Naruemon Bunchom, Weerachai Saijuntha, Ross H. Andrews, Takeshi Agatsuma, Joseph Valencia, Mark June Revolteado, Phoyphaylinh Prasayasith, Pheovaly Soundala, Sonesimmaly Sannikone, Parita Hansana, Marcello Otake Sato, Virasack Banouvong, Philippe Buchy, Moritoshi Iwagami

**Affiliations:** 1 Department of Tropical Medicine and Malaria, National Institute of Global Health and Medicine, Japan Institute for Health Security, Tokyo, Japan; 2 SATREPS Project for Parasitic Diseases (JICA/AMED), Vientiane, Lao PDR; 3 Faculty of Medicine, and Biomedical Science Research Unit, Mahasarakham University, Mahasarakham, Thailand; 4 Center of Excellence in Biodiversity Research, Mahasarakham University, Mahasarakham, Thailand; 5 Department of Surgery & Cancer, Faculty of Medicine, Imperial College, London, UK; 6 Department of Environmental Health Sciences, Kochi Medical School, Nankoku, Kochi Japan; 7 Graduate School of Health Sciences, Niigata University, Niigata, Japan; 8 Immunology Department, Research Institute for Tropical Medicine, Philippine Department of Health, Muntinlupa, Metro Manila Philippines; 9 Institut Pasteur du Laos, Ministry of Health, Vientiane, Lao PDR; 10 Faculty of Medical Technology, Division of Global Environment Parasitology, Niigata University of Pharmacy and Medical and Life Sciences, Niigata, Japan; 11 Center of Malariology, Parasitology and Entomology, Ministry of Health, Vientiane, Lao PDR

**Keywords:** Trematode cercaria, Haplotype network, *cox1*, 28S rRNA, Champasak

## Abstract

**Background:**

Opisthorchiasis, caused by *Opisthorchis viverrini*, is a major public health concern in Southeast Asia. Despite control programs, *O. viverrini* infection persists and contributes to severe liver diseases, including cholangiocarcinoma. This study aimed to assess seasonal variation in trematode prevalence and diversity, evaluate the susceptibility of *Bithynia siamensis* sensu lato lineages II and III to *O. viverrini* infection, and examine the phylogenetic and haplotype network of identified trematode and their snail hosts in Champasak Province, southern Lao PDR.

**Methods:**

Snail samples were collected quarterly in 2024 (February, May, August, and November) from Khong and Mounlapamok Districts using handpicking and scooping. Trematode infections were detected by the crushing method, identified morphologically, and confirmed by molecular analysis. DNA barcoding of nuclear and mitochondrial genes was used to verify trematode species and snail lineages.

**Results:**

Of 1,764 *Bithynia* snails examined, 169 (9.58%) were infected. Five cercarial types were identified: amphistome (3.40%), xiphidiocercariae (2.78%), monostome (2.61%), mixed monostome and amphistome (0.34%), cystophorous (0.28%), and *O. viverrini* (0.17%). Infection rates of *O. viverrini* did not differ between lineages II and III, but other trematodes were significantly more frequent in lineage III (76.67%).

**Conclusions:**

Trematode infection rates and species diversity in *B. s. goniomphalos* show marked seasonal variation in Champasak Province, southern Lao PDR. These findings highlight the complexity of host–parasite interactions and the role of environmental factors shaping transmission, providing insights for targeted prevention and control.

**Graphical Abstract:**

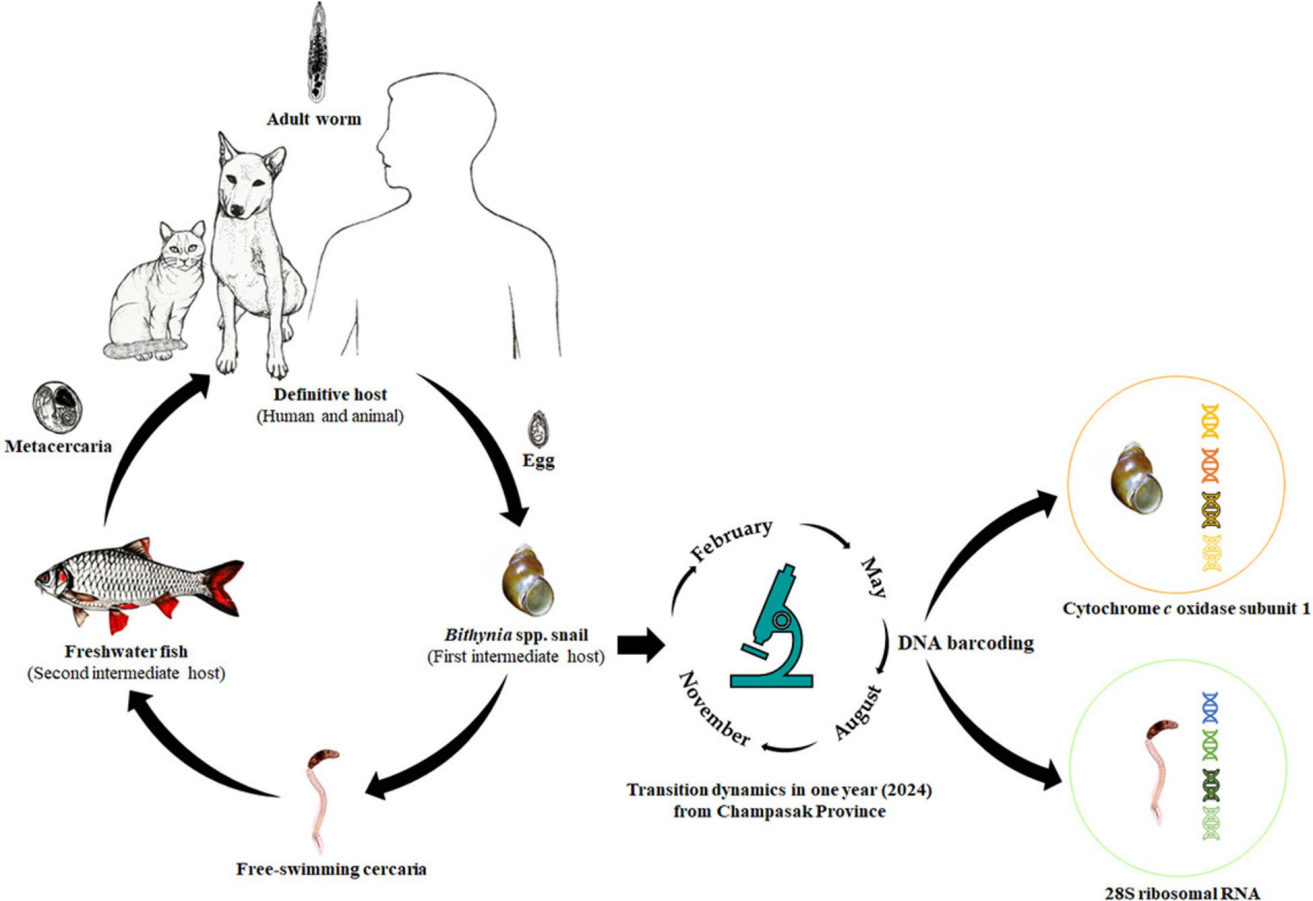

## Background

Freshwater snails of the genus *Bithynia* are widely distributed across Asia and Europe, inhabiting diverse aquatic environments including rivers, lakes, ponds, and rice paddies [1]. Of particular epidemiological importance are *Bithynia siamensis* sensu lato (*B. s. siamensis* and *B. s. goniomphalos*) and *B. funiculata*, which serve as the first intermediate hosts for *O. viverrini*, a medically significant liver fluke endemic to Southeast Asia [2]. This trematode causes opisthorchiasis, a neglected tropical disease closely associated with cholangiocarcinoma (CCA), a lethal bile duct cancer prevalent in countries of the Greater Mekong Subregion, including Thailand, Lao PDR, Cambodia, Vietnam, and Myanmar [3, 4].

Despite extensive research on the epidemiology of *O. viverrini*, infection dynamics within snail hosts require further clarification, particularly in high-transmission areas such as Champasak Province, southern Lao PDR [5, 6]. This region harbors overlapping distributions of two major genetic lineages of *B. s. goniomphalos*, highlighting the potential influence of snail genetic diversity on parasite transmission patterns [7–10]. Although human infection rates are high and cultural practices, such as consumption of raw or undercooked fish, facilitate transmission, comprehensive data on the prevalence, diversity, and seasonal variation of trematode infections in *Bithynia* spp. in this region remain limited.

Traditional methods based on cercarial morphology have been widely used to identify trematode infections in *Bithynia* snails; however, these approaches lack the specificity to differentiate closely related species or co-infections [11–13]. Studies linking morphological identification with molecular confirmation remain sparse, limiting our ability to determine which snail lineages are most susceptible to *O. viverrini* and other trematodes.

Recently, advances in molecular diagnostics, particularly using nuclear markers such as 28S rRNA, have improved the taxonomic resolution of cercariae in snail hosts [7, 8, 14]. Yet, their application in Champasak has been limited. Given the ongoing transmission and ecological complexity of the region, an integrated approach combining molecular identification, lineage-specific susceptibility analysis, and seasonal ecological monitoring is urgently needed.

Understanding how snail–parasite interactions vary across time, space, and genetic backgrounds is essential for predicting transmission risk and informing effective control strategies. This study aims to address these gaps by investigating the prevalence, diversity, and temporal variation of trematode infections in *B. s. goniomphalos* populations in Champasak Province using both morphological and molecular tools. Field surveys were conducted to assess parasite diversity and distribution across multiple sites, enabling the identification of potential transmission hotspots. Cercariae were identified molecularly through phylogenetic analysis of nuclear 28S rRNA sequences, while haplotype network analysis of mitochondrial *cox1* sequences was used to evaluate genetic diversity among infected snails.

## Materials and methods

### Sample collection and identification

**Fig. 1:**
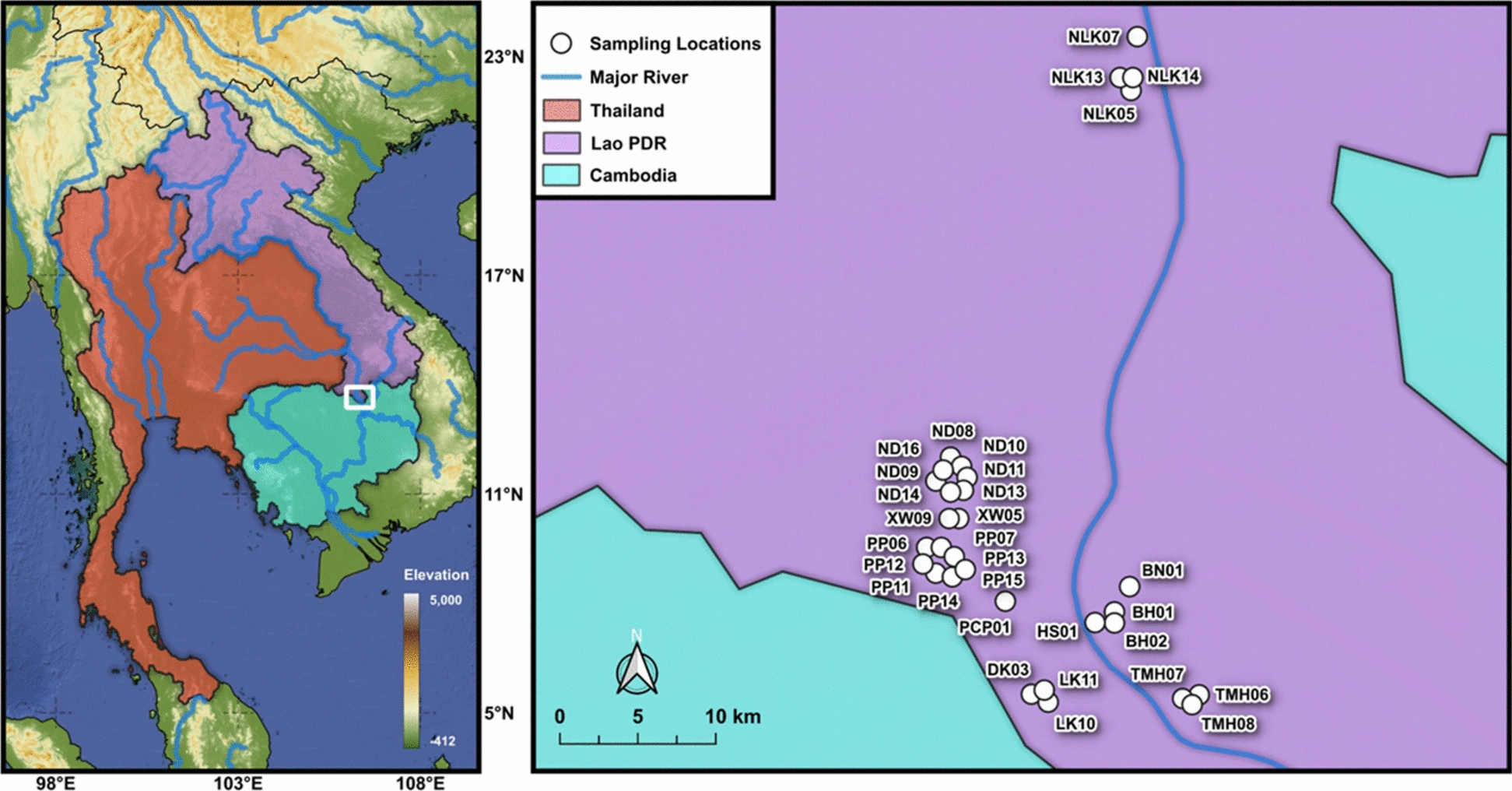
Sampling locations of *Bithynia siamensis goniomphalos* from Champasak Province in Lao PDR. Details of sampling locations are shown in Table 1

In 2024, a total of 1,764 *B. s. goniomphalos* samples were collected quarterly (February, May, August, and November) from 31 sampling locations across eleven villages in southern Lao PDR (Table 1; Fig. 1). The samples were gathered from rice fields and ponds using handpicking and scooping methods, dried with tissue paper, wrapped in newspaper, and subsequently transported to the Institut Pasteur du Laos laboratory for analysis, following the same coding system as Bunchom et al. [15]. Morphological identification was conducted using established keys and descriptions of *Bithynia* species in Southeast Asia, including references by Brandt [16], Upatham et al. [17], Chitramvong [18]. The shells of *B. s. goniomphalos* are small (length ~ 5 mm, width ~ 2 mm), ovately (broadly) conic, and somewhat glossy. The shell surface features thick, raised transverse growth lines and fine spiral lines. The apex is typically intact and not eroded, and the umbilicus is wide and deep, a distinguishing feature. The outer portion of the last whorl is straight, and the basal lip of the aperture lacks a median incision. These characteristics, along with internal anatomical features where needed, were used to confirm species identification and to distinguish *B. s. goniomphalos* from other sympatric bithyniid snails.

**Table 1:** Sampling location, number of samples collected, habitat type, and number of infected snails for each cercarial type of *Bithynia siamensis goniomphalos* from Champasak Province in Lao PDR used in this study

Code	Habitat	Village	District	Latitude	Longitude	Date	*N*	Total number of infected snails	Number of infected snails for each cercarial type						% Infection
									A	B	C	D	E	C + D	
BH01	Rice field	Ban Huay	Khong	14.082769	105.839630	Feb-2024	41	4	–	–	3	1	–	–	1.24 ± 0.37
BH02	Rice field	Ban Huay	Khong	14.082931	105.839105	Feb-2024	42	16	12	–	3	–	1	–	4.95 ± 1.46
PP06	Rice field	Phon Peuy	Khong	14.119862	105.736515	Feb-2024	27	3	1	–	2	–	–	–	0.93 ± 0.26
BN01	Rice field	Ban Na	Khong	14.104553	105.849084	Feb-2024	45	6	4	1	1	–	–	–	1.86 ± 0.48
DK03	Mekong river	Don Khone	Khong	14.040713	105.790748	Feb-2024	9	–	–	–	–	–	–	–	0.00 ± 0.00
HS01	Rice field	Ban Hinsiw	Khong	14.083240	105.828545	Feb-2024	51	13	7	–	2	3	1	–	4.02 ± 0.82
PCP01	Rice field	Phon Champa	Khong	14.095875	105.775275	Feb-2024	100	19	7	–	9	3	–	–	5.88 ± 1.23
XW05	Pond	Xanwa	Mounlapamok	14.145132	105.744332	Feb-2024	8	–	–	–	–	–	–	–	0.00 ± 0.00
						Total	323	61	31	1	20	7	2	0	18.89 ± 2.28
TMH06	Pond	Thamakhep	Khong	14.033899	105.886494	May-2024	7	–	–	–	–	–	–	–	0.00 ± 0.00
TMH07	Rice field	Thamakhep	Khong	14.034484	105.886160	May-2024	26	–	–	–	–	–	–	–	0.00 ± 0.00
TMH08	Rice field	Thamakhep	Khong	14.034488	105.886168	May-2024	71	3	–	–	–	–	3	–	1.83 ± 0.75
PP06	Rice field	Phon Peuy	Khong	14.119862	105.736515	May-2024	5	–	–	–	–	–	–	–	0.00 ± 0.00
LK10	Rice field	Longkang	Khong	14.042361	105.799500	May-2024	22	2	2	–	–	–	–	–	1.22 ± .50
LK11	Pond	Longkang	Khong	14.043139	105.798389	May-2024	16	–	–	–	–	–	–	–	0.00 ± 0.00
ND10	Rice field	Nady	Mounlapamok	14.170972	105.741111	May-2024	5	–	–	–	–	–	–	–	0.00 ± 0.00
ND11	Rice field	Nady	Mounlapamok	14.169806	105.742722	May-2024	5	–	–	–	–	–	–	–	0.00 ± 0.00
XW09	Pond	Xanwa	Mounlapamok	14.145278	105.744806	May-2024	7	–	–	–	–	–	–	–	0.00 ± 0.00
						Total	164	5	2	0	0	0	3	0	3.05 ± 0.69
PP06	Rice field	Phon Peuy	Khong	14.119862	105.736515	Aug-2024	23	2	–	–	–	2	–	–	0.36 ± 0.15
PP07	Rice field	Phon Peuy	Khong	14.125056	105.740306	Aug-2024	93	1	–	–	1	–	–	–	0.18 ± 0.07
PP11	Rice field	Phon Peuy	Khong	14.113000	105.734556	Aug-2024	7	1	–	–	1	–	–	–	0.18 ± 0.07
PP12	Pond	Phon Peuy	Khong	14.118361	105.734556	Aug-2024	66	8	–	–	2	2	–	4	1.43 ± 0.29
PP13	Rice field	Phon Peuy	Khong	14.117750	105.740528	Aug-2024	16	1	–	–	1	–	–	–	0.18 ± 0.07
PP14	Rice field, canal	Phon Peuy	Khong	14.116889	105.743389	Aug-2024	32	3	–	–	1	2	–	–	0.53 ± 0.15
PP15	Pond	Phon Peuy	Khong	14.115722	105.747278	Aug-2024	23	1	–	–	–	1	–	–	0.18 ± 0.07
ND08	Pond	Nady	Mounlapamok	14.171639	105.739389	Aug-2024	38	–	–	–	–	–	–	–	0.00 ± 0.00
ND10	Rice field	Nady	Mounlapamok	14.170972	105.741111	Aug-2024	19	–	–	–	–	–	–	–	0.00 ± 0.00
ND11	Rice field	Nady	Mounlapamok	14.169806	105.742722	Aug-2024	11	2	–	–	–	–	–	2	0.36 ± 0.15
ND13	Pond	Nady	Mounlapamok	14.165167	105.744361	Aug-2024	36	2	–	–	–	2	–	–	0.36 ± 0.15
ND16	Rice field	Nady	Mounlapamok	14.174083	105.736611	Aug-2024	105	3	–	–	2	1	–	–	0.53 ± 0.07
NLK05	Pond	Nangloy Kang	Mounlapamok	14.404445	105.849658	Aug-2024	6	1	–	–	–	1	–	–	0.18 ± 0.07
NLK13	Rice field	Nangloy Kang	Mounlapamok	14.403833	105.851361	Aug-2024	81	5	–	–	–	5	–	–	0.89 ± 0.36
NLK14	Rice field	Nangloy Kang	Mounlapamok	14.403944	105.853694	Aug-2024	5	2	–	–	–	2	–	–	0.36 ± 0.15
						Total	561	32	0	0	8	18	0	32	5.70 ± 0.37
ND08	Pond	Nady	Mounlapamok	14.171639	105.739389	Nov-2024	38	4	–	–	3	1	–	–	0.56 ± 0.17
ND09	Rice field	Nady	Mounlapamok	14.170583	105.740278	Nov-2024	129	9	4	–	5	–	–	–	1.26 ± 0.33
ND10	Rice field	Nady	Mounlapamok	14.170972	105.741111	Nov-2024	110	–	–	–	–	–	–	–	0.00 ± 0.00
ND11	Rice field	Nady	Mounlapamok	14.169806	105.742722	Nov-2024	48	–	–	–	–	–	–	–	0.00 ± 0.00
ND14	Rice field	Nady	Mounlapamok	14.164833	105.744639	Nov-2024	105	3	3	–	–	–	–	–	0.42 ± 0.17
ND16	Rice field	Nady	Mounlapamok	14.174083	105.736611	Nov-2024	55	19	6	2	10	1	–	–	2.65 ± 0.56
NLK05	Pond	Nangloy Kang	Mounlapamok	14.404445	105.849658	Nov-2024	18	12	2	–	–	10	–	–	1.68 ± 0.56
NLK07	Rice field	Nangloy Kang	Mounlapamok	14.430944	105.853611	Nov-2024	10	–	–	–	–	–	–	–	0.00 ± 0.00
NLK13	Rice field	Nangloy Kang	Mounlapamok	14.403833	105.851361	Nov-2024	60	7	1	–	–	6	–	–	0.98 ± 0.34
NLK14	Rice field	Nangloy Kang	Mounlapamok	14.403944	105.853694	Nov-2024	143	17	–	–	–	17	–	–	2.37 ± 0.97
						Total	716	71	16	2	18	35	0	0	9.92 ± 0.98

A = Xiphidiocercariae, B = *Opisthorchis viverrini* cercariae, C = Monostome cercariae, D = Amphistome cercariae, E = Cystophorous cercariae, C + D = Monostome cercariae + Amphistome cercariae

### Examination of *O. viverrini* and other trematodes by crushing method

Each individual snail was crushed between thick glass plates and examined under a stereomicroscope at 10× magnification. This crushing method allows for the identification of various trematode stages, such as rediae, sporocysts, cercariae, and metacercariae, within a short period of time. Trematode parasites were morphologically identified based on the diagnostic characteristics described by Frandsen and Christensen [19]. Trematode cercariae were pooled from a single infected snail host, representing one trematode species, and preserved in 80% ethanol. Additionally, positive head-foot snails were kelp in 80% ethanol for molecular analysis.

### Molecular analysis

Genomic DNA was manually extracted from cercariae and the head-foot tissue of positive snails using the DNeasy^®^ Blood and Tissue Kit (QIAGEN, Germany), following the manufacturer’s instructions. DNA was eluted in a final volume of 100 µl for cercariae and 200 µl for snail tissue. DNA samples were stored at –20 °C until analysis. For detecting and identifying of trematode parasites, the 28S rRNA region (~ 390 bp) was amplified by Polymerase Chain Reaction (PCR) using primers C1 (5ʹ-ACC CGC TGA ATT TAA GCA T-3ʹ) and C3 (5ʹ-CTC TTC AGA GTA CTT TTC AAC-3ʹ) previously described by Lockyer et al. [20]. Each 20 µl PCR reaction included 10 µl of GXL premix (Takara Bio, Japan), 0.4 µl of each primer (10 pmol/µl), 7.2 µl of distilled water, and 2 µl of DNA template (~ 10–20 ng). PCR cycling conditions were: initial denaturation at 30 cycles of 98 °C for 10 s, 56 °C for 15 s, and 68 °C for 20 s.

To identify host snails, a PCR reaction targeting a 710 bp region of the cytochrome *c* oxidase subunit I (*cox1*) of the mitochondrial gene was performed using Folmer's universal primers LCO1490 (5ʹ-GGT CAA CAA ATC ATA AAG ATA TTG G-3ʹ) and HCO2198 (5ʹ-TAA ACT TCA GGG TGA CCA AAA AAT CA-3ʹ) [21]. The PCR amplification protocol consisted of 30 cycles, each comprising denaturation at 98 °C for 10 s, 55 °C for 15 s, and 68 °C for 1 min. PCR products were visualized on a 2% agarose gel using a Mupid^®^-2plus electrophoresis system (TAKARA, Japan) in 1× TAE buffer (Nippon Gene, Japan) at 100 V for 30 min. PCR products were excised and purified using the FastGene™ Gel/PCR Extraction Kit (Nippon Genetics, Japan). Sequencing reactions were performed using the BigDye™ Terminator v3.1 Cycle Sequencing Kit (Thermo Fisher Scientific, USA). Each reaction contained 2.0 µl of 5 × sequencing buffer, 1.0 µl of BigDye™ v3.1, 1.6 µl of primer (1 pmol/µl), 3.4 µl of distilled water, and 2.0 µl of DNA template (~ 150–300 ng of double-stranded DNA), in a total volume of 10 µl. The thermal cycling conditions were as follows: initial denaturation at 96 °C for 2 min, followed by 25 cycles of 96 °C for 10 s, 50 °C for 5 s, and 60 °C for 3 min. Sequencing products were purified using Performa® DTR Gel Filtration Cartridges (EdgeBio, USA), following the manufacturer’s instructions. Purified products were then sequenced using an Applied Biosystems 3130xl Genetic Analyzer (Hitachi, Japan).

### Data analysis

Statistical analyses were performed using Minitab (version 18, Minitab Inc., USA). Means and standard deviations (SD) were calculated using IBM SPSS Statistics (version 19). One-way ANOVA was used to evaluate differences in trematode infection prevalence across sampling periods, locations, and lineages. When significant differences were detected, Duncan’s Multiple Range Test [22] was applied as a post hoc test at a 95% confidence level.

Raw sequence chromatograms were manually checked and edited for base-calling accuracy using BioEdit v7.2.5 [23]. Forward and reverse reads were assembled into consensus sequences in MEGA XI [24]. The edited sequences were compared with reference sequences in GenBank using BLAST on the NCBI platform, showing percentage identities ranging from 95 to 100%, depending on the species or lineage. Sequences were then aligned with GenBank references and included in the phylogenetic analyses. Phylogenetic trees were constructed using both neighbor-joining (NJ) and maximum likelihood (ML) methods in MEGA XI [24]. For the ML analysis, the general time reversible model with gamma distribution and invariant sites (GTR + G + I) [25] was selected, with bootstrap support estimated from 1,000 replicates. In addition, haplotype networks were constructed using the median-joining (MJ) algorithm [26], implemented in Network v5.0.1.1 (https://www.fluxus-engineering.com/).

## Results

### Prevalence of *O. viverrini* cercariae and other trematode infections

**Fig. 2:**
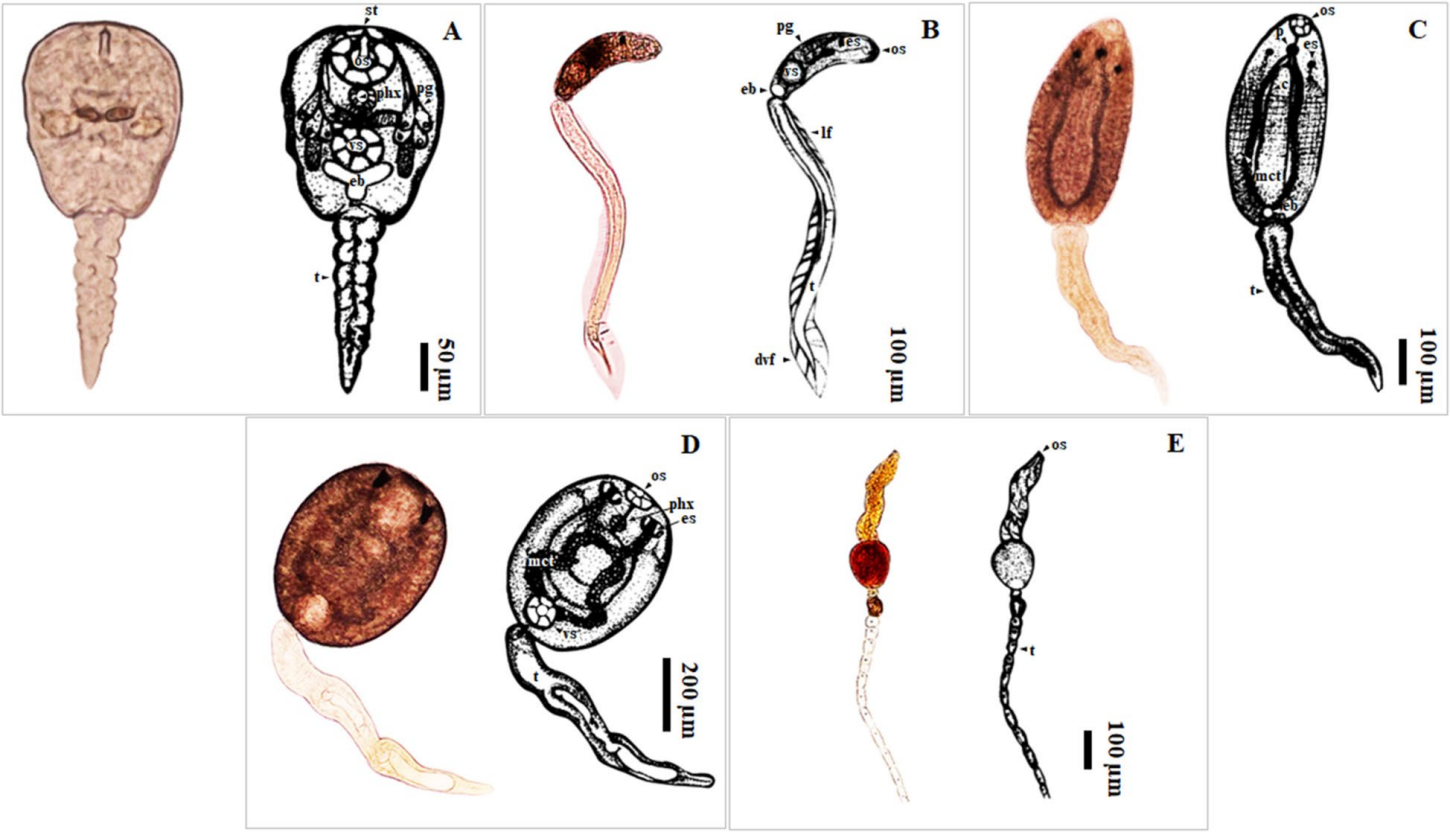
Type of cercariae infecting *Bithynia siamensis goniomphalos* from Champasak Province in Lao PDR. **a** Xiphidiocercaria; **b** *Opisthorchis viverrini* cercaria; **c** Monostome cercaria; **d** Amphistome cercaria; **e** Cystophorous cercaria. c: caecum; dvf: dorsoventral finfold; eb: excretory bladder; es: eyespots; lf: lateral finfold; mct: main collecting tube; os: oral sucker; p: pharynx; pg: penetration gland; phx: phyrynx; st: stylet; t: tail

A total of 1,764 *B. s. goniomphalos* snails were examined for infections with *O. viverrini* and other trematodes. The overall infection prevalence of 169 (9.58%) of snails with five types of cercariae: xiphidiocercaria, *O. viverrini* cercariae, monostome cercariae, amphistome cercariae, and cystophorous cercariae (Table 1; Fig. 2). Additionally, some snails were co-infected with two trematodes: monostome and amphistome cercariae. Among these, amphistome cercariae were the most common (3.40%), followed by xiphidiocercariae (2.78%), monostome cercariae (2.61%), co-infection with monostome and amphistome cercariae (0.34%), cystophorous cercariae (0.28%), and *O. viverrini* cercariae (0.17%) (Table 2).

**Table 2:** The prevalence of trematode infection in *Bithynia siamensis goniomphalos* from Champasak Province in Lao PDR (see Fig. 2 for each cercarial code)

Type of cercariae	Code	Prevalence of infection (%)				Total (*N* = 1,764)	Infected snails (%) in *cox1* lineage	
		February 2024 (*N* = 323)	May 2024 (*N* = 164)	August 2024 (*N* = 561)	November 2024 (*N* = 716)		II	III
Xiphidiocercaria	A	9.60 ± 1.38	1.22 ± 0.41	0.00 ± 0.00	2.23 ± 0.30	2.78 ± 0.82	5.00 ± 0.96	35.00 ± 6.73
*Opisthorchis viverrini* cercariae	B	0.31 ± 0.11	0.00 ± 0.00	0.00 ± 0.00	0.28 ± 0.09	0.17 ± 0.05	3.33 ± 0.89	1.67 ± 0.68
Monostome cercariae	C	6.19 ± 0.96	0.00 ± 0.00	1.43 ± 0.13	2.51 ± 0.41	2.61 ± 0.53	8.88 ± 1.82	21.67 ± 2.45
Amphistome cercariae	D	2.17 ± 0.42	0.00 ± 0.00	3.21 ± 0.25	4.89 ± 0.81	3.40 ± 0.86	3.33 ± 0.61	13.33 ± 2.51
Cystophorous cercariae	E	0.62 ± 0.14	1.83 ± 0.61	0.00 ± 0.00	0.00 ± 0.00	0.28 ± 0.09	0.00 ± 0.00	1.67 ± 0.68
Monostome cercariae + Amphistome cercariae	C + D	0.00 ± 0.00	0.00 ± 0.00	1.07 ± 0.20	0.00 ± 0.00	0.34 ± 0.17	3.33 ± 0.89	3.33 ± 0.86
	Total	18.89 ± 2.28^a^	3.05 ± 0.69^b^	5.70 ± 0.37^b^	9.92 ± 0.98^b^	9.58 ± 1.49	23.33 ± 3.28	76.67 ± 13.49

^a^^, b^Significant interactions (*p* < 0.05) between the seasons

Over the course of the year, 169 out of 1,764 snails (or 9.58%) were infected with trematode cercariae. This figure represents the total infection prevalence across all cercarial types and quarterly months sampled. The overall prevalence of trematode infection in the snail population fluctuated across the year, with the highest infection rates observed in February (18.89%) and the lowest in May (3.05%). August and November showed moderate prevalence, with infection rates of 5.70% and 9.92%, respectively (Table 2). Xiphidiocercaria infects amphibians, which are its definitive hosts. It had the highest infection rate in February (9.60%), with a decrease in May (1.22%) and August (2.23%). *O. viverrini* cercariae typically infect mammals, such as humans, cats, and dogs. Notably, *O. viverrini* infections were relatively rare, with a minimal prevalence in February (0.31%) and November (0.28%) but no infected case was detected in May or August. Monostome cercariae infect birds and fish. This cercaria type showed a higher prevalence during February (6.19%), dropped off in May, then increased in August (1.43%) and November (2.51%). Amphistome cercariae infect ruminant mammals (e.g., buffalo, cattle, and goats). Its prevalence was low in February (2.17%), increased slightly in August (3.21%), peaked in November (4.89%) but was absent in May, reaching a cumulative highest prevalence of 3.40% over the year. Cystophorous cercariae also infect birds and fish, with relatively low infection rates observed. It was present in February (0.62%) and May (1.83%), but not in August or November. Co-infection between monostome and amphistome cercariae, which infect both birds and ruminant mammals was observed primarily in August (1.07%) (Table 2).

### Analysis of phylogenetic relationships between trematode species

**Fig. 3:**
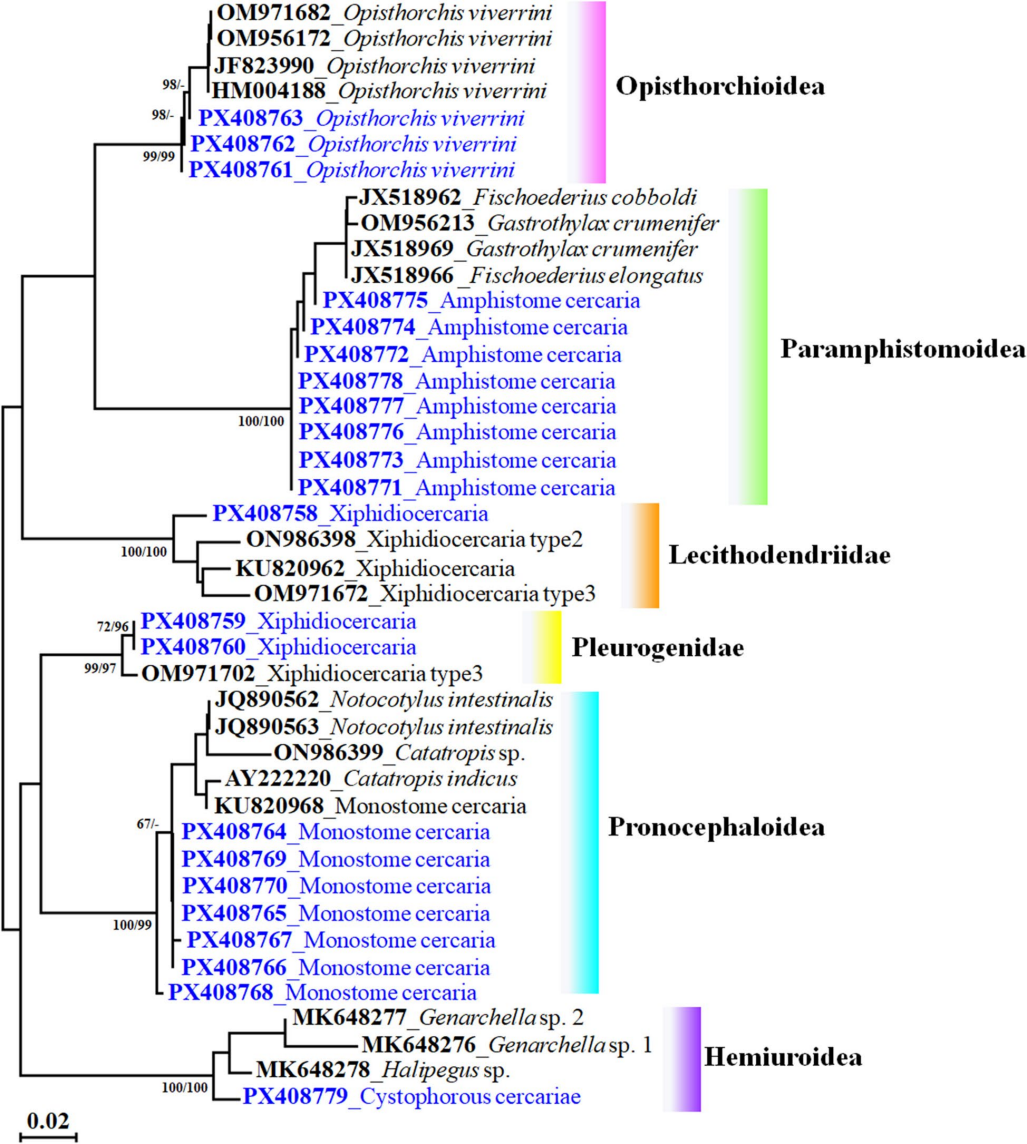
A phylogenetic tree based on partial 28S ribosomal RNA sequences can be used to identify and classify trematode cercariae emerging from *Bithynia siamensis goniomphalos* snails in Champasak Province, Lao PDR

A total of 22 partial 28S rRNA gene sequences (GenBank accession numbers PX408758–PX408779) were obtained, comprising three sequences from *O. viverrini* cercariae and 19 from other trematode species (Table 3). Phylogenetic analysis based on these sequences revealed distinct evolutionary groupings among the five morphologically identified cercarial types. Notably, xiphidiocercariae clustered within the families Lecithodendriidae and Pleurogenidae, both belonging to the superfamily Microphalloidea, indicating a close evolutionary relationship with microphalloid trematodes. *Opisthorchis viverrini* cercariae grouped with both *O. viverrini* and *Clonorchis sinensis*, confirming its classification within the Opisthorchioidea superfamily of liver flukes. Monostome cercariae were placed in the Pronocephaloidea, a lineage associated with bird hosts. Amphistome cercariae aligned with the Paramphistomoidea, consistent with trematodes infecting the digestive tracts of ruminants. Cystophorous cercariae were grouped within the Hemiuroidea, a family commonly associated with aquatic hosts, particularly fish (Fig. 3; Table 3).

**Table 3:** The details of each cercarial type were compared with reference sequences available in the GenBank database to confirm species identification using 28S rRNA gene

Type of cercaria	Isolate	Family	Superfamily	Host (Source)	Definitive host group	Country	GenBank accession number (28S rRNA)	References
Xiphidiocercaria	HS01-C02	Lecithodendriidae	Microphalloidea	*B. s. goniomphalos*	Poultry, Amphibians	Lao PDR	PX408758	This study
Xiphidiocercaria	BN01-C03	Pleurogenidae	Microphalloidea	*B. s. goniomphalos*	Poultry, Amphibians	Lao PDR	PX408759	This study
Xiphidiocercaria	BN01-C10	Pleurogenidae	Microphalloidea	*B. s. goniomphalos*	Poultry, Amphibians	Lao PDR	PX408760	This study
Xiphydiocercariae type 2 (*Lecithodendrium* sp.)	H-28			*B. s. goniomphalos*		Viet Nam	ON986398	[27]
Xiphydiocercariae type 3 (*Paralecithodendrium* sp.)	Xiphy3-Bf			*B. funiculata*		Viet Nam	OM971672	[27]
Xiphydiocercariae type 3 (*Pleurogenidae* sp.)	Xiphy3-Mt			*Melanoides tuberculata*		Viet Nam	OM971702	[27]
Xiphidiocercaria (*Trematoda* sp.)	WN-2016			Freshwater snail		Thailand	KU820962	[28]
*O. viverrini* cercaria	BN01-C45-Ov	Opisthorchiidae	Opisthorchioidea	*B. s. goniomphalos*	Mammals, Rodents	Lao PDR	PX408761	This study
*O. viverrini* cercaria	ND16-C01-Ov	Opisthorchiidae	Opisthorchioidea	*B. s. goniomphalos*	Mammals, Rodents	Lao PDR	PX408762	This study
*O. viverrini* cercaria	ND16-C02-Ov	Opisthorchiidae	Opisthorchioidea	*B. s. goniomphalos*	Mammals, Rodents	Lao PDR	PX408763	This study
*O. viverrini* cercaria	THA-SK	Opisthorchiidae	Opisthorchioidea	*Barbodes gonionotus*		Thailand	JF823990	[29]
*O. viverrini* cercaria	SK			*Puntius brevis*		Thailand	HM004188	[30]
*O. viverrini* cercaria	Pleu1_Bsg			*B. s. goniomphalos*		Viet Nam	OM971682	[27]
*O. viverrini* cercaria	Pleu1_Bf			*B. funiculata*		Viet Nam	OM956172	[27]
Monostome cercaria	BH02-C01	Notocotylidae	Pronocephaloidea	*B. s. goniomphalos*	Mammals, Poultry	Lao PDR	PX408764	This study
Monostome cercaria	BH02-C21	Notocotylidae	Pronocephaloidea	*B. s. goniomphalos*	Mammals, Poultry	Lao PDR	PX408765	This study
Monostome cercaria	BH02-C34	Notocotylidae	Pronocephaloidea	*B. s. goniomphalos*	Mammals, Poultry	Lao PDR	PX408766	This study
Monostome cercaria	ND16-C01	Notocotylidae	Pronocephaloidea	*B. s. goniomphalos*	Mammals, Poultry	Lao PDR	PX408767	This study
Monostome cercaria	PP06-C05	Notocotylidae	Pronocephaloidea	*B. s. goniomphalos*	Mammals, Poultry	Lao PDR	PX408768	This study
Monostome cercaria	PP13-C01	Notocotylidae	Pronocephaloidea	*B. s. goniomphalos*	Mammals, Poultry	Lao PDR	PX408769	This study
Monostome cercaria	PCP01-C09	Notocotylidae	Pronocephaloidea	*B. s. goniomphalos*	Mammals, Poultry	Lao PDR	PX408770	This study
Monostomecercaria (*Trematoda* sp.)	Monostomecercaria WN-2016			Freshwater snail		Thailand	KU820968	[28]
*Catatropis indicus*				*Cairina moschata*		Australia	AY222220	[31]
*Notocotylus intestinalis*	Ni-04			Freshwater snail		Viet Nam	JQ890562	[32]
*Notocotylus intestinalis*	Ni-05			Freshwater snail		Viet Nam	JQ890563	[32]
*Catatropis* sp.	H-45			*B. funiculata*		Viet Nam	ON986399	[27]
Amphistome cercariae	ND11-C01	Gastrothylacidae or Paramphistomidae	Paramphistomoidea	*B. s. goniomphalos*	Ruminants	Lao PDR	PX408771	This study
Amphistome cercariae	NLK05-C01	Gastrothylacidae or Paramphistomidae	Paramphistomoidea	*B. s. goniomphalos*	Ruminants	Lao PDR	PX408772	This study
Amphistome cercariae	NLK05-C02	Gastrothylacidae or Paramphistomidae	Paramphistomoidea	*B. s. goniomphalos*	Ruminants	Lao PDR	PX408773	This study
Amphistome cercariae	NLK13-C01	Gastrothylacidae or Paramphistomidae	Paramphistomoidea	*B. s. goniomphalos*	Ruminants	Lao PDR	PX408774	This study
Amphistome cercariae	PP06-C01	Gastrothylacidae or Paramphistomidae	Paramphistomoidea	*B. s. goniomphalos*	Ruminants	Lao PDR	PX408775	This study
Amphistome cercariae	PP11-C01	Gastrothylacidae or Paramphistomidae	Paramphistomoidea	*B. s. goniomphalos*	Ruminants	Lao PDR	PX408776	This study
Amphistome cercariae	PP12-C01	Gastrothylacidae or Paramphistomidae	Paramphistomoidea	*B. s. goniomphalos*	Ruminants	Lao PDR	PX408777	This study
Amphistome cercariae	PP14-C01	Gastrothylacidae or Paramphistomidae	Paramphistomoidea	*B. s. goniomphalos*	Ruminants	Lao PDR	PX408778	This study
*Gastrothylax crumenifer*	Amphis_Bf			*B. funiculata*		Viet Nam	OM956213	[27]
*Fischoederius elongatus*	Rumen			*Bubalus bubalis*	Ruminants	India	JX518966	[33]
*Gastrothylax crumenifer*	Rumen			*Bos indicus*	Ruminants	India	JX518969	[33]
*Fischoederius cobboldi*	Rumen			*Capra hircus*	Ruminants	India	JX518962	[33]
Cystophorous cercariae	BH02-C31	Derogenidae	Hemiuroidea	*B. s. goniomphalos*	Amphibians, Reptiles, Fish	Lao PDR	PX408779	This study
*Genarchella* sp. 1	257			*Herichthys labridens*		Mexico	MK648276	[34]
*Genarchella* sp. 2	60			*Astyanax aeneus*		Mexico	MK648277	[34]
*Halipegus* sp.	141			*Lithobates* sp.		Mexico	MK648278	[34]

### Haplotype network of infected snail hosts to explore susceptibility patterns

**Fig. 4:**
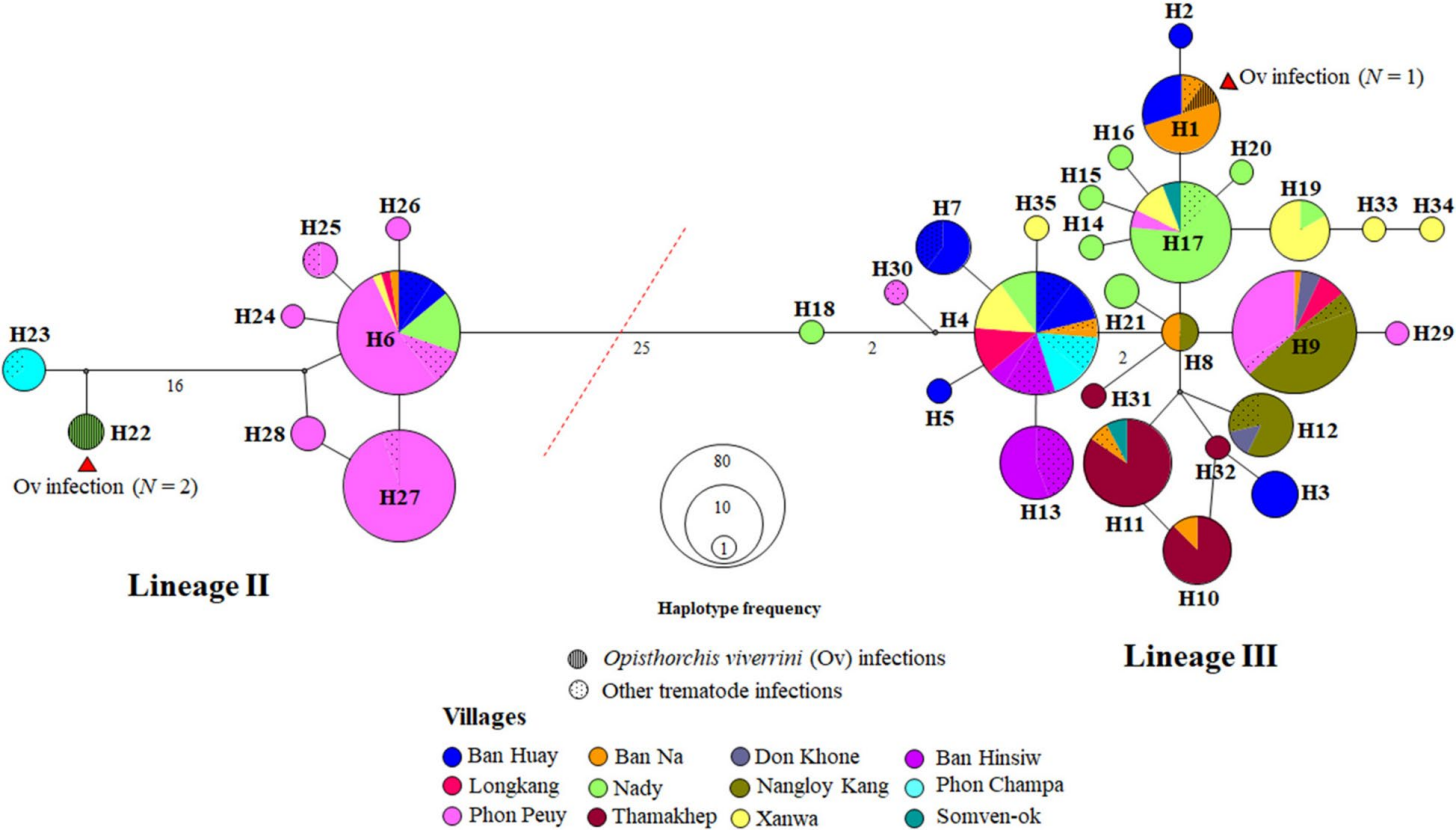
A minimum spanning haplotype network based on mitochondrial *cox1* sequences was constructed to assess the genetic diversity of *Bithynia siamensis goniomphalos* in Champasak Province, Lao PDR. The network, generated using the Median-Joining (MJ) method, reveals variation within the population, with haplotypes represented by circles whose sizes correspond to the number of individuals sharing each haplotype. Mutational steps between haplotypes are indicated along the branches, with one mutational step being shown without a number. Location codes are provided in Table 1 (the same code as Bunchom et al. [15])

A total of 60 *cox1* sequences of *B. s. goniomphalos* (GenBank accession numbers PX398279–PX398338) were analyzed, comprising three sequences from snails infected with *O. viverrini* and 57 from snails infected with other trematodes. The median-joining (MJ) haplotype network constructed from these *cox1* sequences revealed no clear geographic structuring among genetic clusters of infected snails. Individuals from 12 villages across Champasak Province, Lao PDR, were interspersed throughout the network, indicating a high degree of genetic mixing and lack of spatially distinct haplotype groupings. The identified haplotypes were divided into two main lineages, separated by 25 mutational steps. Lineage III was the most prevalent, comprising 27 haplotypes distributed across all villages. Haplotype 4 (H4) was the most dominant, including 80 samples from seven villages—Ban Huay, Ban Na, Ban Hinsiw, Longkang, Nady, Phon Champa, and Xanwa—and was also shared by 29 trematode-infected snails. Haplotype 1 (H1) included one *O. viverrini*-infected and one other trematode infected snail collected from Ban Na (Fig. 4). Lineage II consisted of eight haplotypes identified in samples from Ban Huay, Ban Na, Longkang, Nady, Phon Champa, Phon Peuy, and Xanwa. Within this lineage, haplotype 6 (H6) was the most common, shared by 43 samples: 27 from Phon Peuy, seven from Nady, six from Ban Huay (four of which were obtained in this study), and one each from Ban Na, Xanwa, and Longkang. Additionally, lineage II included two *O. viverrini*-infected snail samples belonging to haplotype 22 (H22), both collected from Nady. These samples were separated by 16 mutational steps from other members of Lineage II (Fig. 4).

Susceptibility patterns for *O. viverrini* infection in *B. s. goniomphalos* showed that lineage II had a slightly higher infection rate (3.33%) compared to lineage III (1.67%) (Table 2). However, other trematode infections were more common in lineage III (76.67%) than in lineage II (23.33%) (Table 2). The highest susceptibility in lineage III was observed with xiphidiocercariae (35.00%), followed by monostome (21.67%), amphistome (13.33%), and co-infection between monostome and amphistome (3.33%). Infections with *O. viverrini* and cystophorous cercariae were both recorded at 1.67% (Table 2).

## Discussion

**Fig. 5:**
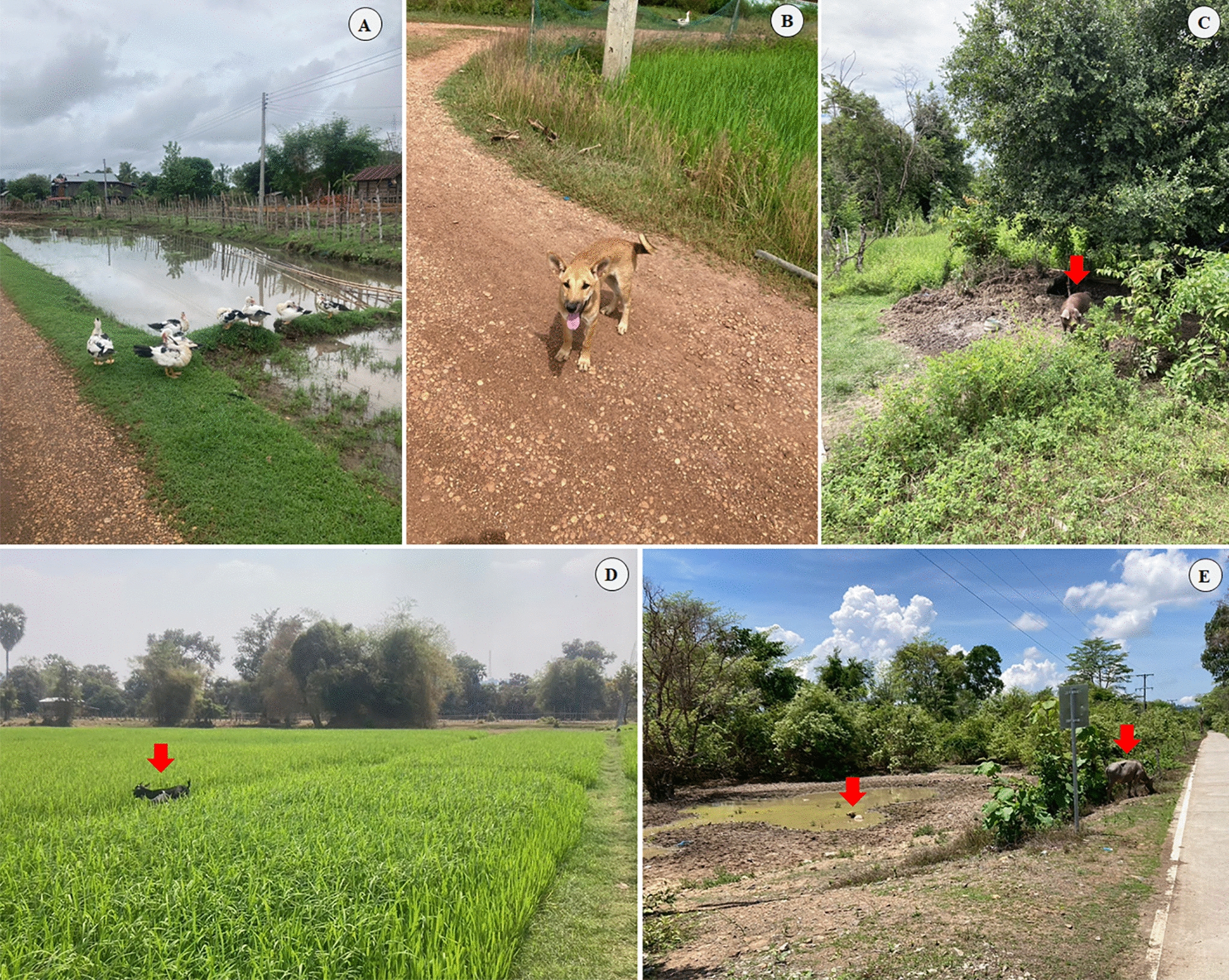
Free-roaming definitive hosts observed in the sampling localities. **a** Free-roaming duck; **b** Dog; **c** Pig; **d** Goat; **e** Buffalo

This study is the first to highlight the prevalence of infection of *B. s. goniomphalos* snails by *O. viverrini* cercariae in Champasak Province, Lao PDR. Although *O. viverrini* infection is already known to occur in humans in this region [5, 6], its detection in *B. s. goniomphalos* snails had never been reported until now. This new data highlights the importance of our study, which also sheds light on the diversity of trematodes in *B. s. goniomphalos* in Champasak Province by combining morphological and molecular approaches to identify the parasites and their snail hosts. Our results revealed five types of cercariae infecting *B. s. goniomphalos*: xiphidiocercariae, *O. viverrini* cercariae, monostome cercariae, amphistome cercariae, and cystophorous cercariae. The overall prevalence of trematode infection in the snail population was 9.58%, with the highest prevalence observed in February 2024. A one-way ANOVA revealed that the differences in prevalence among sampling periods were statistically significant (*p* < 0.05). Notably, amphistome cercariae were the most commonly detected trematode type, consistent with previous studies. During field sampling, we observed various animal populations at the collection sites, including common domestic ruminants such as goats, cattle, and buffalo, as well as ducks, dogs, and pigs (Fig. 5). According to Kiatsopit et al. [12], a total of 20 trematode types of cercariae were identified in Thailand and Lao PDR. The overall prevalence of *O. viverrini* cercariae was 1.59% in Thailand and 0.96% in Lao PDR. These findings suggest that *B. s. goniomphalos* is highly susceptible to infection by a wide range of trematode species. Environmental changes over time, especially seasonal variations, significantly influence host-parasite interactions and infection rates [12, 35, 36]. Infected snails are present throughout the year; however, drought conditions negatively affect their survival, growth, reproduction, and susceptibility to parasitic infection. Prolonged aestivation may contribute to parasite mortality, while the immune response of the snail host plays a key role in the success or failure of parasite infection [37].

*Bithynia* snails show variable infection rates, up to 6.93% in Thailand and 8.37% in Lao PDR [11], with *O. viverrini* cercariae ranking as the fourth most common type in the region. *Bithynia s. goniomphalos* is also susceptible to other trematodes [12, 13, 38]. Despite generally low prevalence, transmission risk remains high due to the large number of cercariae released per infected snail. Our study revealed seasonal and spatial variation in infection rates, with higher prevalence near households and agricultural areas during the rainy season, and *O. viverrini* detected mainly in February and November. No significant differences were observed between lineages II and III. Environmental factors, raw fish consumption, wetland reliance, and ecological disruption contribute to the persistent transmission of *O. viverrini* in the region [13, 39].

However, non-opisthorchiid trematodes remain poorly understood, particularly due to limited molecular data [7, 12, 38]. To date, few studies have employed molecular techniques—such as the use of 28S rRNA and ITS2 genetic markers—for the identification of these trematodes [8]. In our study, we also utilized the 28S rRNA gene to support the molecular identification and classification of trematode species found in infected snail hosts. A phylogenetic tree based on partial 28S ribosomal RNA sequences from different types of cercariae found in *B. s. goniomphalos* snails revealed that the diversity and abundance of the seasonal trematode infection related to landscape epidemiology. For example, in the Lawa Lake region of Khon Kaen Province, Thailand, high abundances of *B. s. goniomphalos* snails and cyprinid fish, key intermediate hosts for *O. viverrini* were observed, especially near the shore and in the lake's southern area during the rainy season. These abundance patterns were linked to nutrient enrichment, salinity, and nitrite-nitrogen levels, indicating that water contamination may influence snail community structure through environmental and species-specific tolerance mechanisms [39].

**Fig. 6:**
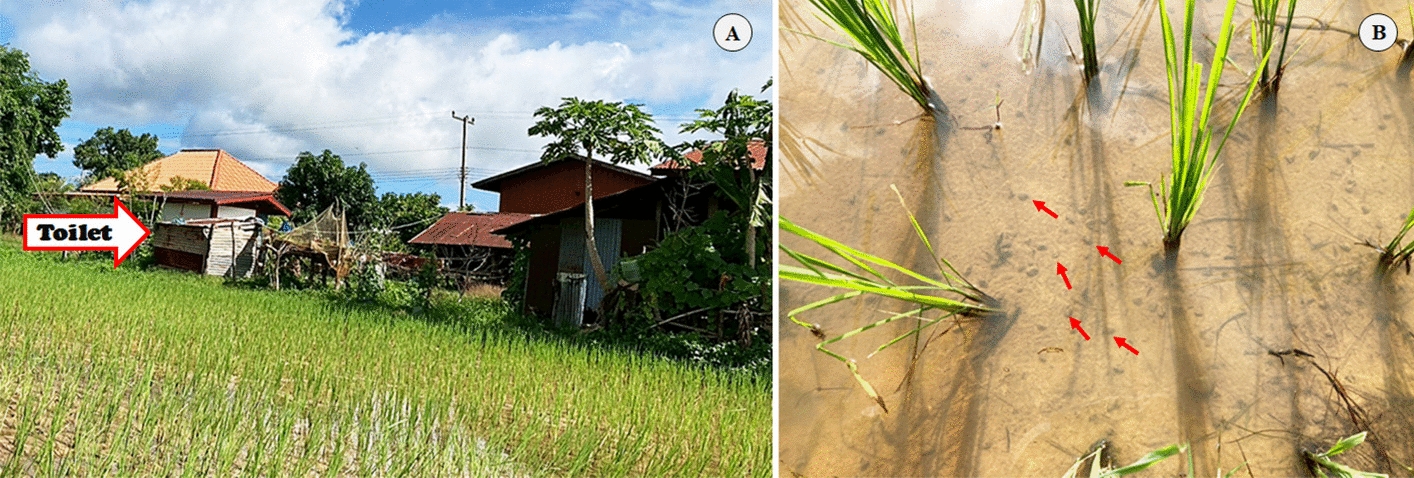
Locality where *Opisthorchis viverrini* infection is found. **a** Household and toilet connected to rice field; **b** *Bithynia* spp. in rice field (red arrows)

*Bithynia s. goniomphalos* has been reported in three Provinces of Lao PDR—Vientiane, Savannakhet, and Champasak—where three distinct genetic lineages (I, II, and III) have been identified [10, 15]. Lineage I is distributed from Vientiane to Savannakhet, whereas lineages II and III are found from Savannakhet to Champasak. In this study, we examined the genetic lineages of infected snail hosts to assess whether lineage is associated with susceptibility to *O. viverrini* infection in natural habitats. Our findings revealed no clear correlation between genetic lineage and susceptibility patterns, suggesting that environmental and ecological factors may play a more significant role in influencing infection dynamics than host genetic background. For example, *O. viverrini* cercarial infection in Nady (ND16) revealed that the area between households and toilets may facilitate the parasite’s life cycle, potentially increasing the likelihood of snail hosts becoming infected (Fig. 6).

A haplotype network based on the *cox1* gene sequences of *B. s. goniomphalos* revealed that lineage II of this snail species is genetically distinct, separated by 16 mutational steps from other lineages (Fig. 4). This genetic separation suggests a long and specific evolutionary history. The findings may support the idea that lineage II of *B. s. goniomphalos* has co-evolved with *O. viverrini*, meaning the parasite and this specific snail lineage have adapted to each other over time. This co-evolution explains why lineage II is more commonly and successfully infected by *O. viverrini* compared to other trematodes; it’s a better biological fit due to shared evolutionary history.

*Opisthorchis viverrini* cercarial infection had a very low infection rate (0.17%) and infected few snails. These data can explain that trematode transmission is more efficient when intermediate and definitive hosts coexist in the same aquatic environment. Similar to the previous report by Bunchom et al. [38], the infection rate is influenced by transmission dynamics related to environmental contamination. Additionally, a high abundance of snails-hosts at a locality can promote cercarial infection, as the reduced distance required for miracidia to locate and infect the hosts increases the likelihood of successful infection [40]. Consequently, more snails-hosts become infected. This could help explain why the site with the lowest snail abundance also exhibited the lowest infection rates—fewer hosts mean reduced opportunities for miracidia to complete their lifecycle [41].

This study has some limitations. Definitive host identification relied on field observations rather than systematic surveys, which may reduce accuracy in estimating host abundance. Morphological identification of cercariae and snails, without full molecular confirmation, carries a risk of misidentification. Phylogenetic analysis was limited by the quality and availability of reference sequences in public databases, potentially affecting the robustness of inferred relationships. In addition, small sample sizes in some subgroups may restrict the generalizability of the findings. Future studies addressing these limitations will improve understanding of host–parasite dynamics in this region.

## Conclusion

This study highlights the role of *B. s. goniomphalos* in the transmission of trematodes affecting both humans and animals, offering insights for more targeted surveillance and control in endemic areas. However, infection patterns varied across regions and seasons due to environmental factors like temperature, drought, salinity, and land use. Long-term monitoring is essential to understand how these factors influence snail-host susceptibility and parasite transmission. Additionally, limited molecular data on snail and reservoir hosts point to the need for further research into their genetic diversity and role in sustaining transmission cycles.

## Data Availability

The datasets used and/or analyzed in the current study are available from the corresponding author upon reasonable request.

## Abbreviations

*cox1*: Cytochrome *c* oxidase subunit 1
28S rRNA: 28S ribosomal RNA
16S rRNA: 16S ribosomal RNA
ITS2: Internal transcribed spacer2
*N*: Number of *B. s. goniomphalos* examined
H: Haplotype
NJ: Neighbor-joining
ML: Maximum likelihood
MJ: Median-joining
